# Biparental Care in Insects: Paternal Care, Life History, and the Function of the Nest

**DOI:** 10.1673/031.013.13101

**Published:** 2013-11-21

**Authors:** Seizi Suzuki

**Affiliations:** Ecology & Systematics, Graduate School of Agriculture, Hokkaido University, Sapporo 060-8589, Japan

## Abstract

The evolution of parental care is a complex process, and many evolutionary pathways have been hypothesized. Maternal care is common, but paternal care is not. High confidence of paternity should favor the evolution of paternal attendance in caring for young; biparental care is rare because paternity assurance is typically low compared to maternity. Biparental care in insects has evolved several times and has high diversity. To evaluate the conditions for the evolution of biparental care, a comparison across taxa is suitable. In this review, common traits of biparental species are discussed in order to evaluate previous models of biparental care and the life history of insects. It will be shown that nesting is a common feature in biparental insects. Nest structure limits extra-pair copulations, contributing to the evolution of biparental care.

## Introduction

Parental care is defined as “any form of parental behavior that appears likely to increase the fitness of the parent's offspring” (Clutton-Brock 1991), or “any parental trait that enhances the fitness of a parents' offspring, and that is likely to have originated and /or to be currently maintained for this function” (Royle et al. 2012). An understanding of the evolution of parental care is of central importance in evolutionary biology. Notably, which gender cares for the young is an important question that has stimulated numerous theoretical and empirical efforts (Maynard Smith 1977; Zeh and Smith 1985; Clutton-Brock 1991). Biparental care of offspring is present across the animal kingdom, but the study of parental care has been biased toward birds, cichlid fishes, and primates (Clutton-Brock 1991; Cockburn 2006). In addition, because biparental care in vertebrates is widespread and has evolved only a few times, especially in birds, there have been a limited number of comparative studies (Burley and Johnson 2002).

To identify the conditions favoring the evolution of biparental care, comparisons of common life history traits across the different taxa are instructive for estimating what types of environmental conditions are needed to evolve such common traits. Among insects, parental care is hypothesized to have evolved in more than 10 orders (Tallamy and Wood 1986; Costa 2006). While female care for offspring is relatively common in insects, male contributions to care are rare (Tallamy 1994). Insect biparental care has evolved several times independently. Trumbo (1996) noted that biparental care is associated with nests, and emphasized the importance of phylogenetic comparisons and the comparative physiology of offspring care. Although nest-making has been discussed (Eickwort 1981; Trumbo 1996), the common traits and the values of nests have not been explained in detail.

In this review, I hypothesize that some types of nests and biparental care in insects are correlated, because the nest functions to prevent extra-pair copulation. A comparative test focusing on the role of the male and the type of nest is evaluated in light of the proposed and previous models of biparental care.

## Previous hypotheses of biparental Care

A model using game theory by Maynard Smith (1977) is well-known for explaining the evolution of parental care. However, this model assumed that the fitness of males and females is equal and that there is no division of labor. Additional factors must be considered for the evolution of biparental care.

The effect of paternity on males' care of offspring has been the subject of considerable discussion. If sperm competition exists, males are less certain of their parentage, and this uncertainty of parentage would favor male desertion (Trivers 1972). Westneat and Sherman (1993) suggested that confidence of paternity should favor the evolution of facultative paternal care. Queller (1997) pointed out two reasons why males provide less care than females do. First, multiple mating and sperm competition create uncertainty about paternity for males, diminishing the expected fitness gain of caring for young. Second, a subset of males may be consistently more likely to mate than others if their traits are favored by sexual selection. Wright (1998) advocated a whole mating system approach to the study of paternity and paternal care, and in most cases a lower probability of parentage for males does tend to make males less likely than females to provide care. Houston and McNamara (2002) emphasized the effect of interactions between males and females, and hypothesized the relationships between paternity and the parental effort.

**Table 1.**
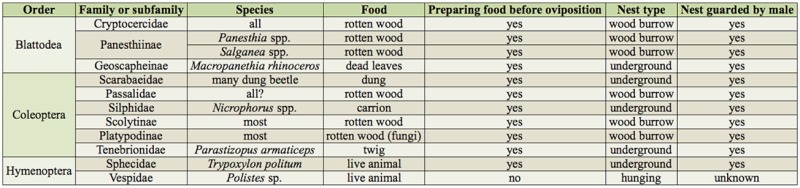
The relationships of taxonomic group showing biparental care and their nest feature.

Wade and Shuster (2002) reanalyzed the Maynard Smith (1977) model. According to the model proposed by Wade and Shuster (2002), if deserting males can gain extra offspring, male parental care evolves whenever half the magnitude of the indirect genetic effect of paternal care on offspring viability exceeds the direct effect of additional mating success gained by desertion. Kokko and Jennions (2008) reviewed the sex roles regarding offspring care, and they suggested that the adult sex ratio will generate differences in breeding systems. Loss of paternity will be generated by female-biased adult sex ratios (e.g., female multiple mating), and it will drive female-only care.

There have been empirical reviews of paternal and/or biparental care of arthropods. Zeh and Smith (1985) provided an overview of three categories (prezygotic paternal investment, biparental care, exclusive paternal care) by terrestrial arthropods, and emphasized that paternal investment is correlated with certainty of paternity and male territoriality. Tallamy (1994) proposed the enhanced fecundity hypothesis, in which paternal investment will evolve as a trait increasing mating opportunities by the benefit of care. Especially when the resources for reproduction are difficult to acquire or digest, paternal attendance tends to evolve because of reduced future reproduction. These studies take note of sexual selection, particularly paternity, but the effect of paternity remains controversial (Kokko and Jennions 2003; Alonzo 2010).

## Biparental care in insects

Biparental care in insects has been reported in three orders: Blattodea, Coleoptera, and Hymenoptera (Table 1). Most species in these orders make nests underground or in wood burrows and prepare food for young in the nest before oviposition is finished. In addition, nest-guarding behavior by males against other males has been reported in most of the species.

### Blattodea

Subsociality has been found among many cockroach species, and there is considerable variation in care, such as tending and protecting their young, feeding young on body fluids, and progressive provisioning (Nalepa and Bell 1997). Biparental care is found in all Cryptocercidae species and some members of Panesthinae and Geoscapheinae in Blaberidae.

**Cryptocercidae.** Cryptocercids are wood feeders, living as family colonies in burrows made in rotting logs. Adults provide defense of the nest and feeding of the young, their hindgut fluids being food for the young (Nalepa 1984; Nalepa and Bell 1997). Colonies with a male and a female pair are common in this group (Nalepa 1984). Both the male and the female participated in an attack against an intruder (Park and Choe 2003a). Nymphs grew more rapidly when cared for by two parents rather than one (Park and Choe 2003b).

**Blaberidae.** Behavioral research has focused on the wood-feeding species of genera *Panesthia* and *Salganea* in the subfamily Panesthinae. Multifemale groups are common in the genus *Panesthia* (O'Neil et al. 1987). Male-male aggression is common, and antagonistic behavior is mainly restricted to adult males (O'Neil et al. 1987). In contrast, with few exceptions, all studied species of *Salganea* live in biparental families consisting of a male-female pair together with their offspring (Maekawa et al. 2008). Like *Cryptocercus*, adults of *Salganea* species defend young nymphs. Parental feeding of the initial instars via stomodeal trophallaxis was observed, and is likely to be important for survivorship and normal growth in the genus (Shimada and Maekawa 2011).

Parental behavior of the subfamily Geoscapheinae has been studied in only one species, *Macropanethia rhinoceros* (Rugg and Rose 1991; Matsumoto 1992). The adult *M. rhinoceros* mate in the burrow and provide their young with leaf litter and frass collected by both sexes (Rugg and Rose 1991). They show a division of labor between the males and females. Adults exhibit two different behaviors, wandering and foraging. “Wandering” is predominantly observed in adult males, and “foraging,” the collection of litter, is predominantly observed in adult females (Rugg and Rose 1991). Males disperse or die earlier than the females.

Subsociality has been found in the species of the families Cryptocercidae and Blaberidae. All species of Cryptocercidae show biparental care. This family has a rather ancestral position in Blattoidea, and the superfamily is not phylogenetically supported (Maekawa and Matsumoto 2000). In contrast, subfamilies Panesthinae and Geoscapheinae are sister groups and apomorphic in Blaberidae (Maekawa et al. 2003). Although other (ancestral) subfamilies of Blaberidae include subsocial species, all of the studied species exhibited maternal care (e.g., Bhoopathy 1998, *Thorax porcellana* of Epilamprinae; Roth 1981, *Perisphaerius semilunatus* of Perisphaeriinae; Grandcolas 1993, *Thanatophyllum akinetum* of Zetoborinae). These results suggest that maternal care is ancestral and biparental care is an apomorphic trait in Blaberidae.

### Coleoptera

Coleoptera is the largest order, with more than 300,000 species. Because of such diversity, many subsocial species are found. The order includes many biparental species (Halffter 1991), which have evolved in at least five families: Scarabaeidae, Passalidae, Silphidae, Tenebrionidae, and Curculionidae.

**Geotrupidae and Scarabaeidae.** Superfamily Scarabaeoidea is an enormous group, especially in light of the “dung beetles,” who use dung as food for their young. Geotrupidae, in the subfamily Scarabaeinae (Scarabaeidae), includes many subsocial species that exhibit biparental care.

Biparental care in dung beetles of Scarabaeinae is common, but the extent of care varies greatly from species to species. All dung beetles bury dung underground (nidification; Halffter and Edmonds 1982). In addition, post-ovipositional care occurs in the species in the tribes Coprini and Oniticellini (Halffter 1997). The destruction of the brood ball by intruder males is found in some species (Halffter et al. 1980). In *Scarabaeus catenatus*, fights between males over a nest are common; fights between females are observed but are less common (Sato 1998).

There have been many phylogenetic studies of the dung beetle (Philips et al. 2004; Monaghan et al. 2007; see also Scholtz et al. 2011). Although monophyly in some tribes is not supported (Scholtz et al. 2011), rough phylogeny is in accord with the hypothesis of the evolution of nesting behavior proposed by Halffter and Edmonds (1982). This hypothesis suggested there is a spectrum ranging from little or no bisexual cooperation with simple nests to complex biparental cooperation with post-ovipositional maternal care.

**Passalidae.** Most passalids live in rotting wood (Schuster 1992). They occur in family groups including male and female parents, eggs, larvae, pupae, and teneral and mature offspring (Schuster and Schuster 1997). Passalid beetles show complex parental care (Schuster and Schuster 1997), including sophisticated feeding behavior. All species in Passalidae show biparental care, but detailed studies of social behavior have been conducted for only a few species. Passalid beetles do not show a division of labor (Valenzuela-González 1993). Although the intensity of aggression in field conditions has not been reported, high levels of aggression between same-sex passalids (Valenzuela-González 1986) and infanticide by intruders were reported in a laboratory study (King and Fashing 2007). Aggression occurs only when immature young are present in the colony, and more frequently against the same sex (Schuster and Schuster 1985).

**Silphidae.** The complex biparental care of burying beetles (Silphidae: *Nicrophorus*) is well-known and has received considerable scientific attention (reviewed in Eggert and Müller 1997; Scott 1998). *Nicrophorus* exploit small vertebrate carrion as food for their young. Typically, a male-female pair prepares a carcass by burying it, removing hair, and rounding it into a ball. Eggs are laid in the soil adjacent to the carrion ball. After hatching, larvae crawl to the carrion ball, where they are fed by parental regurgitations.

*Nicrophorus* are generally monogamous (Trumbo 1992; Trumbo and Eggert 1994; Eggert and Sakaluk 2000) and display intense intrasexual competition in both sexes (Otronen 1988; Suzuki et al. 2005). Both the males and females defend their carcass and brood even after the larvae hatch, by attacking intruders cooperatively (Scott 1990; Robertson 1993; Trumbo 2007). Intruder burying beetles often kill resident larvae, and such infanticide is a regular occurrence in the wild (Scott 1990; Koulianos and Schwarz 2000). Male presence repels intruders irrespective of the sex of the intruders (Trumbo 2006). Burying beetles often breed as male-female pairs, the females doing more feeding and nest maintenance, and the males more guarding (Fetherston et al. 1990; Smiseth and Moore 2004). Brood guarding is the only parental task performed more commonly by males than females.

The underground nesting of *Nicrophorus* reduces the number of intruders (Suzuki 1999). The nest has the function of protection for conspecific intruders. The threat of infanticide by conspecific intruders is thought to be the primary explanation for extended biparental care in burying beetles (Trumbo 2006). The adults of both sexes repel intruders by direct fight, and the underground nests reduce the possibility of intrusion.

The subfamily Nicrophorinae includes three genera: *Nicrophorus*, *Eonecrophorus*, and *Ptomascopus*. *Ptomascopus* and *Nicrophorus* are thought to form a monophyletic group, with *Ptomascopus* apparently retaining more ancestral traits than *Nicrophorus* (Peck and Anderson 1985; Dobler and Müller 2000; Szalanski et al. 2000). *Ptomascopus* exploit small vertebrate carrion like *Nicrophorus*, but do not bury or round the carcass, or feed its larvae with it (Trumbo et al. 2001). *Ptomascopus* parents show a simple and possibly primitive form of parental care (Suzuki and Nagano 2006). *Ptomascopus* males guard their carcasses against other males until oviposition, but rarely guard after hatching (Suzuki et al. 2006). These reports also suggested that *Ptomascopus* females show care for their young, but males show no care or less care.

**Tenebrionidae.** Although the family Tenebrionidae constitutes about 19,000 described species, the desert beetle, *Parastizopus armaticeps*, is the only species in this family known for subsocial behavior. They are usually monogamous, and the malefemale pairs work together to dig a breeding burrow in the sand and fill the burrow with twigs, the food for their offspring. The parents remain inside with the pupal cocoons until the teneral adults eclose (Rasa 1990).

*P. armaticeps* shows an highly specific division of labor between males and females. Females forage on the surface at night for high-quality detritus. Males dig and extend breeding burrows, and they dig the burrows deeper to keep the burrow moist (Rasa et al. 1998). The males primarily guard the burrow entrance against intra- and inter-specific intruders (Rasa and Endrody-Younga 1997; Heg and Rasa 2004).

Tallamy (1994) mentioned the neotropical *Phrenaptes* sp. as being biparental insects, referring to Ohaus (1909). However, Ohaus (1909) observed only two pairs of this species with larvae on the underside of rotten wood, and did not report whether parental behavior was common. It cannot be said whether this behavior is biparental care or not.

**Curculionidae.** In the narrow sense, Curculionidae contains few subsocial species. Wassell (1966) reported both sexes of *Tentegia ingrata* were observed in underground nests containing larvae in dung pellets, and Jordal et al. (2011) mentioned male and female *Homoeometamelus* sp. excavate nuptial chambers by boring wood, and a single egg is laid in each niche. However, neither of these studies described help for the young by the parents, and thus did not confirm whether these species display biparental care or not.

The subfamilies Scolytinae and Platypodinae contain most of the subsocial species in Curculionidae. They live and breed in the tissue of woody plants, mostly in the inner bark (Kirkendall et al. 1997). Most bark and ambrosia beetles construct tunnel systems (sometimes referred to as galleries) in the breeding material. Their mating system is classified in one of four ways: femaleinitiated monogamy, male-initiated monogamy, inbreeding polygyny, or harem polygyny (Kirkendall 1983). Females always stay in the tunnel with their brood, and males often stay as well.

Except for species exhibiting inbreeding polygyny, all types of social structures in bark beetles show male participation in the care of young. Males assist their mates by expelling frass and by defending the gallery against intrusion by insect predators (Reid and Roitberg 1994). Male residency increases the total number of eggs laid in a gallery (Robertson 1998), and competition between mates for access to females is often fierce in bark beetles (Kirkendall 1983). However, Reid and Roitberg (1994) rejected the hypothesis that male residency has a function of mate-guarding, because some removed males were not replaced. Guarding by males has the function of repelling other species (predators), but will have little function in repelling consexual intruders.

The ancestral mating system of bark and ambrosia beetles is thought to be femaleinitiated monogamy (Kirkendall 1983). In most species of bark beetles, each female initiates her own gallery, and a male joins later. Male attendance in offspring care may be a derived trait, though maternal care has not been observed in this group except for in the groups with inbreeding polygyny.

### Hymenoptera

Although Hymenoptera species are well known for their highly structured sociality, there have been a few reports of biparental care. Their haplodiploidy could account for both the tendency toward eusociality (compared with diploid insects) and for the overwhelming tendency for eusocial hymenopteran workers to be female. Male workers are rare in the social Hymenoptera.

However, a few exceptions exist. Males of some species in Sphecidae guard females' nests against conspecific males and parasites. Alcock (1975) and Brockmann and Grafen (1989) studied this behavior and reviewed male guarding behavior in Sphecidae. The male nest-guarding is thought to have evolved from territorial behavior (Alcock 1975). Feeding the young was also reported; Makino (1993) described male feeding young in *Polistes jadwigae*. Hunt and Noonan (1979) reported males of *Polistes fuscatus* and *Polistes metricus* (Vespidae) fed their young, and they reviewed male feeding behavior in Vespidae (found in ten species of Vespidae). However, these examples of feeding by males included food provisioning not by fathers, but by brothers, and the total proportion of feeding was small.

## Discussion

Phylogenetic information regarding insects is incomplete, and more information should be collected to help estimate the origin of biparental care. Some groups seem to evolve maternal care primarily (e.g., Blaberidae, Silphidae), and some show mainly biparental care first (e.g., dung beetle, bark and ambrosia beetle). Although exclusive paternal care has evolved independently in some Heteroptera species (Tallamy 2000, 2001), there is no biparental species in Heteroptera, and there seems to be no groups in which paternal care evolved first. These findings suggest female attendance in an ancestor is needed for the evolution of biparental care.

Alonzo (2010) noted that male care decreased with decreased paternity in less than half of the past studies. Tallamy (2000, 2001) hypothesized that paternal care can evolve the sexual selection of males with superior genes, and females can use nest construction or the act of guarding another female's eggs as honest signals of paternal intent and quality. In addition, the model proposed by Alonzo (2011) showed that female choice for males allows male care to evolve despite low relatedness between the male and the offspring. However, these studies showed evolutionary conditions not in biparental care, but in paternal care. Another explanation is for why males attend to young in the presence of maternal care.

Although biparental behavior varies among taxa, some common traits are found, as shown in Table 1. Food has been considered to be a mover for biparental care because some types of food are difficult to eat for young, such as rotten wood, or are difficult to defend from competitors without help by parents, such as carrion and dung (Tallamy and Wood 1986; Tallamy 1994). It is worth noting that all species of biparental Blattodea, Coleoptera, and Sphecidae of Hymenoptera make nests in the food of the young (e.g., wood-feeding cockroach) or carry the food to their nests before larval hatching (e.g., burying beetle). Some aspects of nests are common among biparental insects: for example, (1) the nest has enough food for young or consists of food itself before finishing oviposition, (2) females usually stay in the nest, and (3) the nest has a tough wall made by soil or wood, which limits entrance. Almost all biparental Blattodea and Coleoptera live in either rotten wood or in an underground nest. Most species finish collecting food before oviposition, and species collecting food after oviposition is rare (Rasa 1990; Rugg and Rose 1991).

Paternity has been assumed to be a prerequisite for the maintenance of biparental care (Westneat and Sherman 1993; Queller 1997; Wade and Shuster 2002; Kokko and Jennions 2003), but few studies have examined the relationship between paternity and biparental care in insects. Extra-pair copulations impede the evolution of biparental care. The presence of “sneaker” males of *Onthophagus taurus* reduces the mass of provisioning and increases the rate of desertion by the paternal males, who are also observed to increase the proportion of time spent guarding females (Hunt and Simmons 2002); thus, male care by *O. taurus* affects not only offspring size but also confidence of paternity. A similar example was reported in *Nicrophorus* (Suzuki 1999). If the nests of most biparental insects have the function of protecting against intrusion by other males, as in *Nicrophorus*, this type of nest will increase the confidence of paternity and promote biparental care for males. If securing paternity promotes biparental care and nest-making, *Polistes* can be regarded as the exception. Because the majority of species of social ants, bees, and wasps mate only once (Strassmann 2001), most males of these species have no need to be cautious about extrapair copulation. This does not contradict the hypothesis of paternity, but why *Polistes* has not adopted this type of the nest is uncertain.

A relationship between nest building and paternity assurance has been reported in some fish species. Nest building in biparental sand gobies, *Pomatoschistus minutus*, has the function of protecting against sneaking by other males (Svensson and Kvarnemo 2003). Kvarnemo (2005) hypothesized that nest building in gobies seems to be important for defense not only against egg predators, but also against sneaker males. In this review, the function of nest building in biparental insects is also to assure paternity. However, because of the internal fertilization of insects, a male must guard not only the nest but also the female, and will elongate guarding the female until oviposition in order to assure its paternity, at which time it will be more necessary for nest guarding. In many biparental insects, food for their young is prepared before oviposition. Because females usually stay in their nests during oviposition, extra-pair copulation is prevented to some extent.

Because the presently observed biparental insect species seem to have evolved from maternal or biparental species, the ancestral species may have faced a rather male-biased operational sex ratio. In such a sex ratio, exclusive paternal care tends not to be evolved (Kokko and Jennions 2008). Under such conditions, mate guarding can enhance male reproductive success. The major function of male care is nest guarding, and some species of females can raise their young without male attendance if there is no intruder (e.g., Trumbo 2006). Even in the biparental species for which male care is necessary for the growth for young, division of labor is often present, and the male task is usually biased to nest guarding (e.g., Rasa 1990). In addition, male *Nicrophorus* beetles show developed care, including provisioning, only guarding against conspecific intruders appears to improve the survival rate of young and thus enhance male reproductive success (Eggert et al. 1998; Trumbo 2006). Nest cleaning by male bark beetles does not enhance the total number of offspring, but it does enhance paternity assurance (Lissemore 1997). Both examples indicate that male care, except nest guarding, does not improve the survival or production of young even when the males show elaborate care other than guarding. In this nest structure of biparental insects, males can be needed to guard females in the nest containing young. In this scenario, females must maintain care because male care, except nest guarding, may not improve their young's survival. If mate guarding enhances not only male reproductive success but also the survival rate of the young, biparental care may evolve easily after mate guarding. If such mate guarding extends beyond oviposition, it will reduce additional mating with other females. In order to evolve additional investments in the young, a benefit for the male is needed. The benefit of male care in the initial condition of young is still unknown. However, male presence in some species can reduce the intrusions of rival males and predators (e.g., Rasa 1990). Thus, it is possible that mate guarding is the initial condition to male attendance in biparental care.

In this paper, it was assumed that the nest of biparental insects limits the access of other males, increases the confidence of paternity, increases the reproductive success for males, and promotes male attendance to care. For example, *Nicrophorus* species show extended biparental care and make a nest, while the sister genus *Ptomascopus* shows maternal care and does not make a nest (Trumbo et al. 2001; Suzuki and Nagano 2006). It is possible that nesting and biparental care have coevolved; however, there have not been enough studies of the function of nests to confirm this hypothesis. Comparative studies of the relation between nest type and paternity are needed, as are studies of nest type and paternity in ancestral (maternal) species. If the hypothesis is correct, the paternity of biparental species will be found to be much higher than in other species. In addition, information on the presence of mate guarding in ancestral species will be important, as will experimental manipulations of the confidence of paternity (e.g., Hunt and Simmons 2002).

A link between biparental care and nest building in insects has been suggested (Eickwort 1981; Trumbo 1996), but the reason why nest building enhances biparental care has not explained. Maternal care will depend on the food supply (Tallamy and Wood 1986), but male attendance will depend not only on food, but also on paternity assurance. A nest that is surrounded by a wall of wood or soil will have the function of preventing extra-pair copulation in biparental insects. If confidence of paternity promotes male attendance of care, this type of nest will increase paternity and promote biparental care.
